# Supplementary material for: Unfolding the multiscale structure of networks with dynamical Ollivier-Ricci curvature
Source: Nat Commun. 2021 Jul 27;12:4561. doi: 10.1038/s41467-021-24884-1 (PMC8316456; doi:10.1038/s41467-021-24884-1)
Supplement: Supplementary file 1 — Supplementary Information [file 41467_2021_24884_MOESM1_ESM.pdf]

Supplementary material to "Unfolding the multiscale structure of networks with dynamical Ollivier-Ricci curvature"

Supplementary Note 1: Classical Ollivier-Ricci edge curvature

The classical formulation[1] of Ricci curvature on metric spaces is a generalisation of the Ricci curvature of differential geometry. Supplementary Fig. 1a illustrates the construction of the classical Ricci curvature.

We consider two points x and y on a manifold, a vector $\mathbf{v}$ on the tangent plane at x which is parallel transported along the geodesic connecting x and y to obtain the vector $\mathbf{v}'$. These vectors shift x and y to nearby points x' and y', which will be at a distance $d_{x'y'} \approx d_{xy}(1 - ||\mathbf{v}||^2 K_w/2)$, where K_w is the sectional curvature. The Ricci curvature Ric_{xy} between points x, y is then defined as the average sectional curvature and is proportional to

$$Ric_{xy} \propto 1 - \frac{\langle d_{x'y'} \rangle}{d_{xy}}\,, \tag{1}$$

where $\langle \cdot \rangle$ denotes the average over all vectors $\mathbf{w}, \mathbf{w}'$ over the unit sphere in the tangent planes at x and y. In other words, the Ricci curvature quantifies the average expansion or contraction of geodesics around the points x and y. On flat spaces the geodesics stay equally separated hence $Ric_{xy} = 0$, on compact spaces the geodesics contract hence $Ric_{xy} > 0$, while on hyperbolic spaces they expand resulting in $Ric_{xy} < 0$.

The classical Ollivier-Ricci curvature[1] is defined as a discrete analogue of this construction. For any two nodes i and j, the averages over local vectors $\mathbf{v}$ and $\mathbf{v}'$ are replaced by local measures (one-step probability measures) $\mathbf{p}_i = \delta_i \mathbf{K}^{-1}\mathbf{A}$ and the Ollivier Ricci curvature is defined as

$$\kappa_{ij} = 1 - \frac{\mathcal{W}_1(\mathbf{p}_i, \mathbf{p}_j)}{d_{ij}}\,. \tag{2}$$

We remark that Supplementary Eq. (2) and the dynamical OR curvature in Eq. (1) are valid for any two nodes i, j if the denominator is replaced by the geodesic distance between i and j, but for this work, it suffices to consider adjacent nodes. Indeed, for any non-adjacent nodes uv, $\kappa_{uv} \geq \kappa_{u'v'}$, where $u'v'$ is an adjacent pair lying on the geodesic connecting u, v (Proposition 19 in Ref.[1]), meaning that local curvatures control global curvatures.

Supplementary Note 2: Expected Laplacian spectrum of the symmetric stochastic block model

In this note, we compute the spectrum of the expected normalised Laplacian matrix of the symmetric SBM $\mathcal{G}(n,\, k_{\mathsf{in}}/n,\, k_{\mathsf{out}}/n)$. Here k_{in} and k_{out} are constants representing the expected number of edges within and across clusters, respectively. The expected adjacency matrix of the symmetric stochastic block model is:

$$\langle \mathbf{A} \rangle_{\mathcal{G}} = \begin{pmatrix} \frac{k_{\mathsf{in}}}{n}\mathbf{1}_{n/2\times n/2} & \frac{k_{\mathsf{out}}}{n}\mathbf{1}_{n/2\times n/2} \\ \frac{k_{\mathsf{out}}}{n}\mathbf{1}_{n/2\times n/2} & \frac{k_{\mathsf{in}}}{n}\mathbf{1}_{n/2\times n/2} \end{pmatrix}. \tag{3}$$

Then, the expected normalised Laplacian matrix is given by

$$\langle \mathbf{L} \rangle_{\mathcal{G}} = \mathbf{I} - \begin{pmatrix} \frac{2k_{\mathsf{in}}}{n(k_{\mathsf{in}}+k_{\mathsf{out}})} \mathbf{1}_{n/2\times n/2} & \frac{2k_{\mathsf{out}}}{n(k_{\mathsf{in}}+k_{\mathsf{out}})} \mathbf{1}_{n/2\times n/2} \\ \frac{2k_{\mathsf{out}}}{n(k_{\mathsf{in}}+k_{\mathsf{out}})} \mathbf{1}_{n/2\times n/2} & \frac{2k_{\mathsf{in}}}{n(k_{\mathsf{in}}+k_{\mathsf{out}})} \mathbf{1}_{n/2\times n/2} \end{pmatrix} \tag{4}$$

The first eigenvector is $\phi_1 = \mathbf{1}_n/\sqrt{n} \in \mathbb{R}^n$, with the corresponding eigenvalue being $\lambda_1 = 0$. The second eigenvector has two values, one on each cluster of the SBM. Taking $\phi_c(u) = 1/\sqrt{n}$ for $1 \leq u \leq n/2$ and $-1/\sqrt{n}$ for $n/2 < u \leq n$ one has asymptotically

$$\langle \mathbf{L} \rangle_{\mathcal{G}} \phi_c = \left[1 - \frac{k_{\mathsf{in}}}{k_{\mathsf{in}} + k_{\mathsf{out}}} \frac{2}{n} - \frac{k_{\mathsf{in}}}{k_{\mathsf{in}} + k_{\mathsf{out}}} \frac{2}{n} \left(\frac{n}{2} - 1 \right) + \frac{k_{\mathsf{out}}}{k_{\mathsf{in}} + k_{\mathsf{out}}} \frac{2}{n} \frac{n}{2} \right] \phi_c \xrightarrow{n\to\infty} \frac{2k_{\mathsf{out}}}{k_{\mathsf{in}} + k_{\mathsf{out}}} \phi_c. \tag{5}$$

Supplementary Note 3: Clustering benchmarks on generative networks

We compare the performance of geometric clustering to standard clustering algorithms for the Stochastic Block model (SBM)[2] and Lancichinetti-Fortunato-Radicchi (LFR)[3] generative benchmark graphs. In each case, we draw a graph from the respective generative models and compute the curvature at a set of discrete τ within an interval (see Supplementary Tables 1, 2 for parameters). Then, as described in the main paper, we perform 10^2 Louvain runs to optimise the weighted and signed modularity of the curvature weighted graph at each timestep. We compute the variation of information between Louvain clusterings at each scale and detect the best scale by finding the minimum of the variation of information values across scales. We then compare the identified clustering with the ground truth based on the adjusted Rand index[4].

In the SBM benchmark, graphs were generated from the symmetric two-partition SBM. To test the limits of the algorithm we have set the average degree to be $\bar{k} \ll n$ in order to generate graphs in the sparse regime (see Supplementary Fig. 2 and Supplementary Table 1 for parameters). We then varied r, the ratio of between to within edges, relatively to the Kesten-Stigum limit r_{KS} below which efficient cluster detection is not possible[5]. Clustering in the sparse regime is particularly hard because the graph communities do not contain a 'core', which can be captured by density-based algorithms. Geometric modularity performed close to the theoretical (Kesten-Stigum) limit given by the belief propagation method[5,6] (Supplementary Fig. 2a). As expected, geometric clustering outperformed classical node clustering methods including spectral clustering[7] and Girvan-Newman[8] (edge betweenness), which only perform well when the graph is dense. Remarkably, geometric modularity also significantly outperformed modularity[9], confirming that it is the edge curvatures which contain the cluster structure and not density differences, which could otherwise be captured by modularity alone. We found that the method of Ni et al.[10] using Ricci flow based on the classical Ollivier-Ricci notion failed to cluster the graph for any value of r. This suggests that in the sparse regime, diffusions allow to average out fluctuations. We note that the difference between belief propagation and geometric modularity are likely due to finite size effects or the stochasticity of the Louvain algorithm. In particular, in the main paper we show that the spectrum of the diffusion process pair that we use to construct the edge curvature has a dominant eigenvector which approximates the true cluster structures (Fig. 3). However, this result is only true asymptotically and we had to simulate graphs of up to 10^5 nodes to obtain a good numerical match (Fig. 3c).

The LFR benchmark is a generalisation of the SBM where the number of clusters and the number of nodes within clusters are exponential random variables. As a consequence, we only tested geometric modularity against methods that do not require specifying the number of sought after clusters a priori. We find that geometric modularity performs as well as the state-of-the art classical modularity and spinglass methods and substantially better than the Ricci flow method[10]. The fact that geometric modularity performs as well as standard modularity is a statement that information is not lost by reweighting the edges by the curvatures and that the correct scale τ can indeed by identified from the variation of information of clustering obtain from multiple Louvain runs.

Supplementary Note 4: Comparison of clustering algorithms on *C. elegans* homeobox gene network

In Supplementary Fig. 3, we compare the clustering for the *C. elegans* dataset obtained by geometric modularity (Fig. 4, main text) with several other clustering methods taken from the cdlib library (`https://doi.org/10.5281/zenodo.5035974`), the Markov Stability method[11] and the wavelet method of Tremblay and Borgnat[12] (see Supplementary Table 3 for the list of methods). To assess the quality of clustering, we compute the variation of information (VI) against the ground truth, as in the main text, as well as the number of detected clusters. We observe that most methods under-perform on this example because either they are bound to finding large-scale clusters or prone to overfitting by finding too many clusters (Supplementary Fig. 3a). Only the geometric modularity method introduced herein could detect the correct community scale (Fig. 4g in the main text). In Supplementary Fig. 3b, we computed the clustering obtained from the wavelet method[12] for a range of scales (note that the convention of the scale used therein is different from the scale used in this paper). The best scales were identified from the local maxima in the stability function (Supplementary Fig. 3b, orange line). The quality of these scales was then assessed by computing the VI against the ground truth partition (Supplementary Fig. 3b, blue line). Based on maxima of the stability, the wavelet method finds a plateau of large-scale structures and a stable scale near the best possible clustering (scale 2, blue vertical bar). We also mark the scale of the best clustering obtained by the wavelet method (scale 1), although note that this scale is not indicated by the maxima in the stability, and therefore would not be identified by the method.

Supplementary Note 5: Computational complexity

In this section we perform a rough empirical assessment of the geometric modularity clustering algorithm. The computational complexity of our clustering method is determined by four components: (i) the computation of the geodesic distances, (ii) the computation of the diffusion measures (Eq. (1)), (iii) the computation of the optimal transport distance (Eq. (11)) and (iv) the computation of the clustering. First, the geodesic distances are computed using the Floyd-Warshall algorithm[13] which runs in $O(n^3)$ time. Second, we compute each diffusion measure in Eq. (1) by the scaling and squaring algorithm of Ref.[14]. To our knowledge, the complexity of this algorithm is not known, but we can parallelise the faster evaluation of the matrix exponential acting on each delta, $\delta_i e^{-\tau \mathbf{L}}$, function rather than computing the matrix exponential $e^{-\tau \mathbf{L}}$ and then multiplying by δ_i. Third, the exact computation of Eq. (11) is performed by the interior point method implementation (taken from `https://pythonot.github.io/`) which has a complexity $\mathcal{O}(n^{5/2})$[15]. Hence evaluating on all edges requires $O(mn^{5/2})$. Fourth, the clustering is performed by the Louvain algorithm[9], which for sparse graphs runs in time $O(n)$.

From the above analysis we see that the computation of the curvatures and possible the diffusion measures place the largest demands on the complexity of our algorithm. As a baseline, to get an aggregate cost of computing (ii)-(iii), we measured the time for computing curvatures for all edges of a set of ER graphs with various node degrees n and edge probabilities $p = c/n$ for some constant c. Supplementary Fig. 4a shows that the computation time scales approximately as $O(m^2) < O((n^2 c/n)^2) = O(n^2)$ in the number of nodes and $O(m)$ in the number of edges. This confirms that the curvature computation has complexity approximately $O(mn^2)$, which is close to the theoretical estimate of $O(mn^{5/2})$.

In our implementation the computation of (ii) and (iii) are highly parallelised and we implement various measures to reduce computational cost. For small times τ the measures $\mathbf{p}_i$, $\mathbf{p}_j$ concentrate in the vicinity of their starting nodes i, j. In such cases one may trim the measures by setting p_i^k, p_j^k to zero whenever they are less than some cutoff c. This substantially speeds up the computation while retaining a good numerical accuracy (Supplementary Fig. 4b). After trimming the complexity is only slightly larger than $O(m)$, which means the support size of the measures stays close to constant as the number of nodes increases. For larger times, the cutoff can still be applied but may have negligible effect if most nodes support a mass large enough. However, since the explicit computation of transport plan $\boldsymbol{\zeta}(\tau)$ in the optimal transport distance

(Eq. (11)) is not required, one may use the Sinkhorn distance[16] to approximate the optimal transport problem. This involves adding an entropic regularisation term

$$\begin{aligned} &\mathcal{W}_1(\mathbf{p}_i(\tau),\, \mathbf{p}_j(\tau)) = \min_{\boldsymbol{\zeta}} \sum_{uv} d_{uv}\zeta_{uv} + \gamma \sum_{uv} \zeta_{uv} \log \zeta_{uv}\,, \\ &\text{subject to } \sum_{v} \zeta_{uv} = p_i^u(\tau)\,, \quad \sum_{u} \zeta_{uv} = p_j^v(\tau)\,. \end{aligned} \tag{6}$$

The regularised Sinkhorn distance approximates the exact solution for small enough γ, while permitting near $\mathcal{O}(n)$-time computation of the optimal transport distance[16]. Being an iterative method, the Sinkhorn distance can also be computed on GPU; an option that we have implemented based on the Python Optimal Transport package[17]. This yields a computational complexity of $\mathcal{O}(nm)$ for sparse graphs (Supplementary Fig. 4c). However, we warn the reader that the magnitude of the regularization term γ required to produce results close to the exact computation are dependent on the number of nodes with larger graphs requiring smaller γ. This need to be addressed on a case by case basis by progressively decreasing γ until the results become stable.

Supplementary Figures

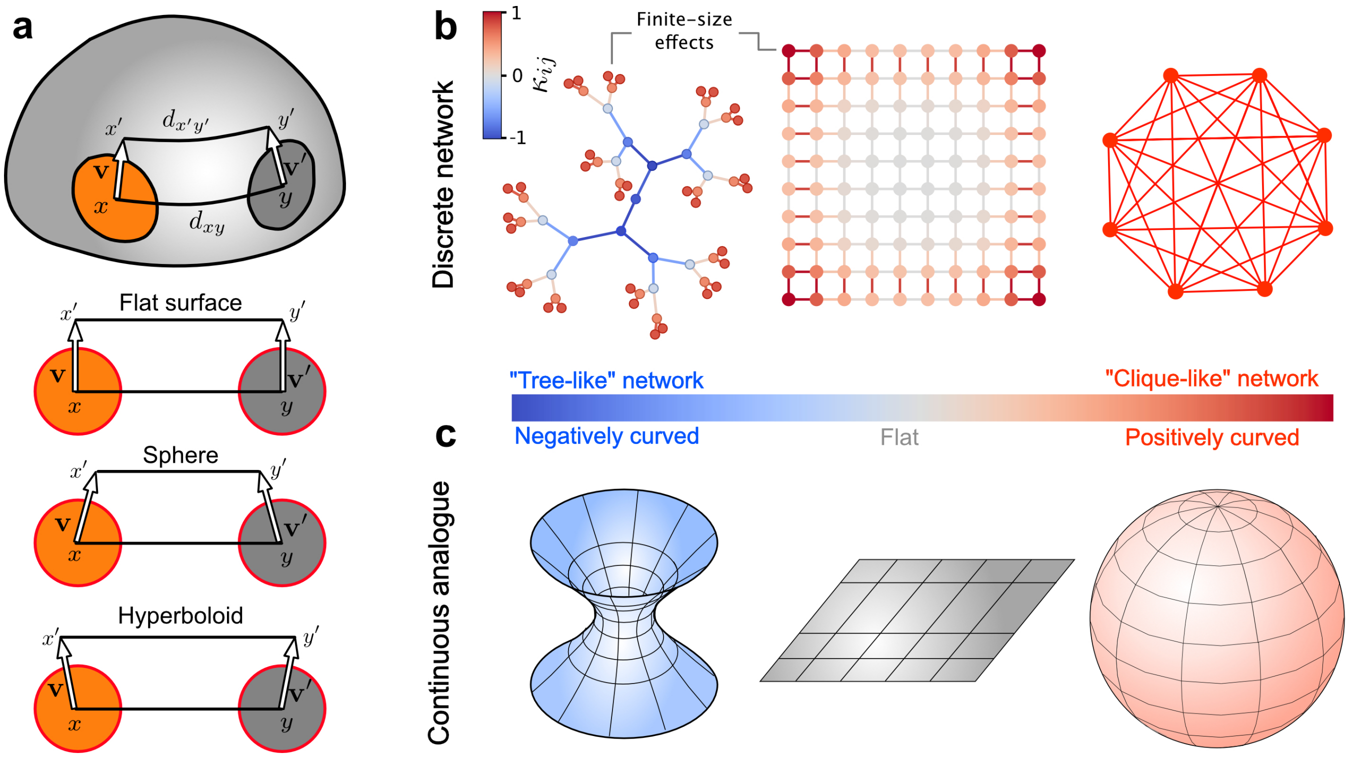

Supplementary Figure 1: Ricci and dynamical Ollivier Ricci curvature on canonical surfaces and graph structures. **a** Ricci curvature on a manifold. The geodesic distance of close points x and y on average changes when translated by parallel vectors $\mathbf{v}$ and $\mathbf{v}'$ on the unit circle in the tangent planes at x and y. On planes the points remain equidistant, on spheres the points contract and on hyperbolic surface they expand. **b** Canonical graphs with edges coloured by the dynamical OR curvature (Eq. (1)) for $\tau = 1$ show that positively and negatively curved graphs have qualitatively different topologies. Away from the boundaries, tree-like topologies are negatively curved, grid-like topologies are flat (zero curvature), whereas clique-like topologies attain positive curvature. Nodes are coloured by the average edge curvature across the neighbours. **c** Analogously, the differential geometric notion of Ricci curvature is negative on hyperbolic surfaces, zero on planes and positive on spherical surfaces.

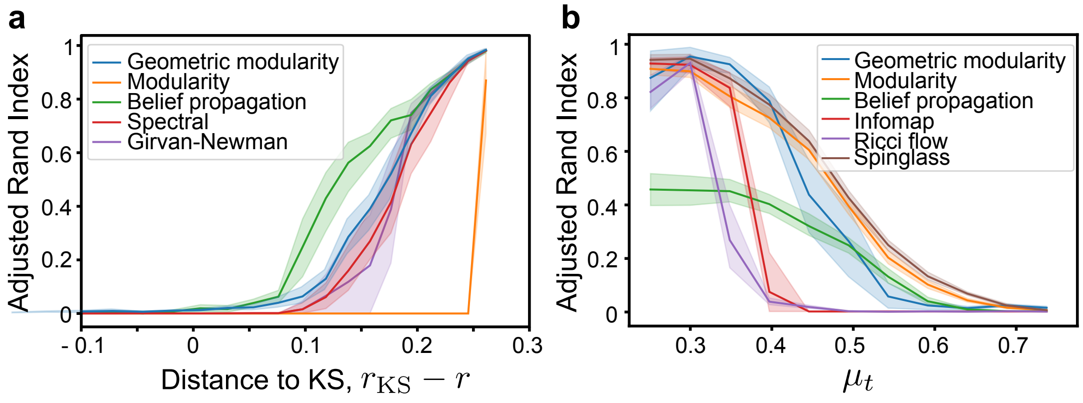

Supplementary Figure 2: Comparison of geometric modularity with state-of-the-art unsupervised clustering algorithms Clustering performance on the **a** symmetric sparse SBM graph ($\bar{k} = 3$, $n = 1000$) **b** LFR benchmark ($n = 500$, $\tau_1 = 3$, $\tau_2 = 1.5$, $\bar{k} = 20$, $k_{\mathsf{min}} = 20$, $k_{\mathsf{max}} = 50$, number of communities between 10 and 50).

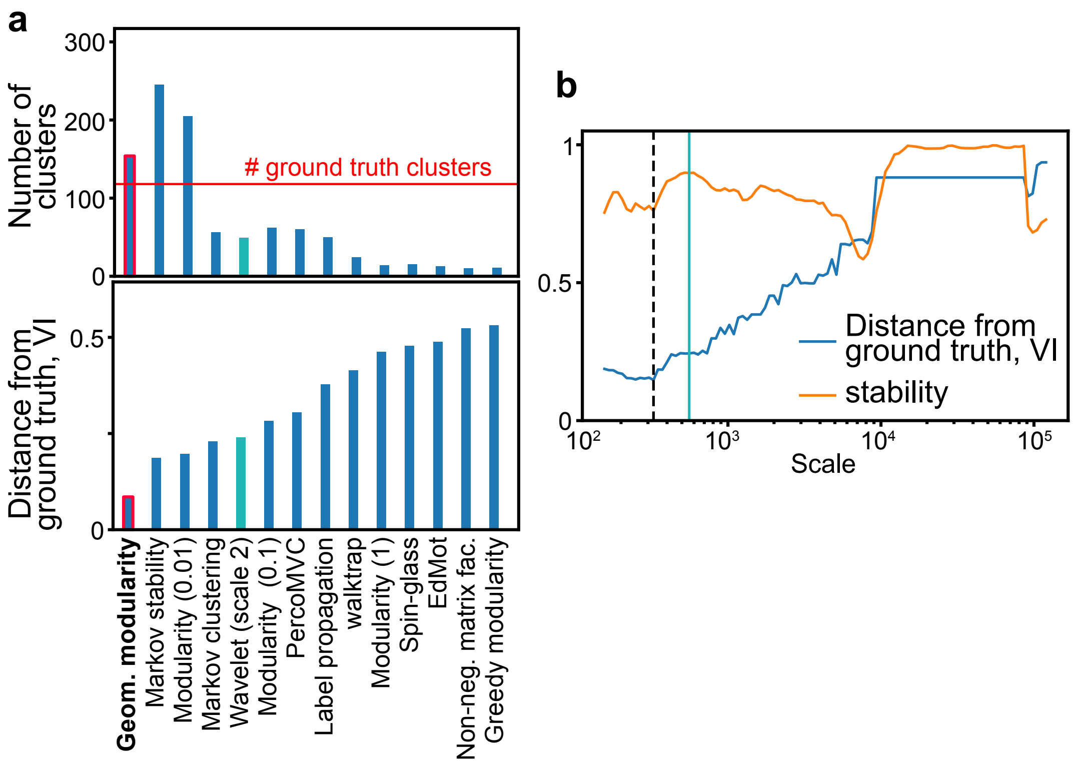

Supplementary Figure 3: Clustering algorithms applied on the *C. elegans* homeobox gene network. Performance of geometric modularity compared with several algorithms from the cdlib library as well as Markov stability, Louvain and wavelet clustering[12] (see Supplementary Table 3 for parameters). **a** Number of clusters and distance to ground truth of the best clustering identified by the respective methods. Green bar corresponds to the best scale identified by the wavelet method shown in **b**. Red box indicates the best scale found by the geometric modularity method. **b** Stability of clustering scales obtained by the wavelet clustering method of Tremblay and Borgnat[12]. Blue vertical line indicates the location of the local stability maximum closest to the ground truth. Dashed vertical line indicates the clustering that is closest to the ground truth, but with no corresponding maxima in stability and thus cannot be identified.

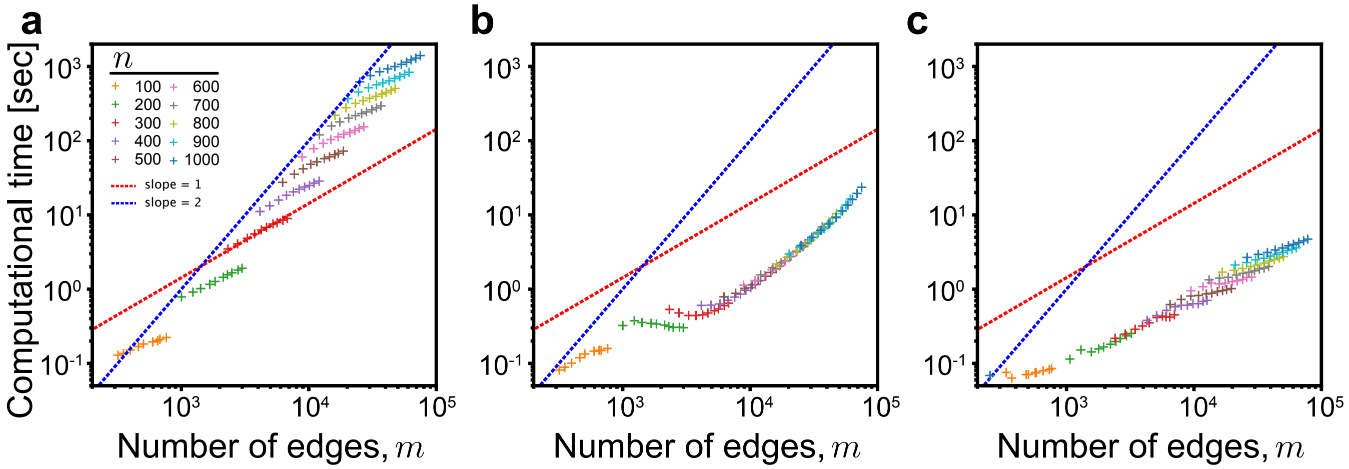

Supplementary Figure 4: Complexity of the curvature computation for Erdős-Rényi graphs Complexity of the curvature computation on ER graphs of various sizes (n) and densities (p). **a** Computational time of the exact optimal transport distance against number of edges. **b** Computational time for the exact optimal transport distance when the measures are trimmed below a cutoff of 10^{-4}. **c** Computational time of using the Sinkhorn distance ($\gamma = 1$).

Supplementary Tables

Supplementary Table 1: Clustering algorithms used for the SBM benchmark

**Method**	**Source**	**Parameters**
Geometric modularity	this paper	$-0.5 < log_{10}\tau < 0$, κ_0=0
Modularity	[9]	Resolution parameter = 1
Belief propagation	[18]	Max. iterations=100, Tolerance=10^{-4}, Re-runs=5, Threshold=0.005, q_{max}=7
Spectral	[7]	Number of clusters = 2
Girvan-Newman	[8]	Level to cut the dendrogram = 1

Supplementary Table 2: Clustering algorithms used for the LFR benchmark

**Method**	**Source**	**Parameters**
Geometric modularity	this paper	$-0.5 < log_{10}\tau < 0$, κ_0=0
Modularity	[9]	Resolution parameter = 1
Belief propagation	[18]	Max. iterations=100, Tolerance=10^{-4}, Re-runs=5, Threshold=0.005, q_{max}=7
Spectral	[7]	Number of clusters = 40
Infomap	[19]	
Ricci-flow	[10]	Number of iterations = 100
Spinglass	[20]	

Supplementary Table 3: Clustering algorithms used for the *C. elegans* homeobox gene network

**Method**	**Source**	**Parameters**
Geometric modularity	this paper	$-0.5 < log_{10}\tau < 0$, κ_0=0
Modularity	[9]	Resolution parameter = = 1, 0.1, 0.01
Wavelet	[12]	$2 < s < 5$
Markov stability	[11]	$-0.5 < log_{10}\tau < 0$
Markov clustering	[21]	expansion=2, inflation=2, loop=1, iterations=100, threshold=0.001
PercoMVC	[22]	
Label propagation	[23]	
walktrap	[24]	
Spinglass	[20]	
EdMot	[25]	
Non-negative matrix factorisation	[26]	
Greedy modularity	[27]	

Supplementary References

[1] Ollivier, Y. Ricci curvature of Markov chains on metric spaces. *J. Funct. Anal.* **256**, 810–864 (2009).

[2] Holland, P. W., Laskey, K. B. & Leinhardt, S. Stochastic blockmodels: First steps. *Soc. Netw.* **5**, 109–137 (1983).

[3] Lancichinetti, A. & Fortunato, S. Benchmarks for testing community detection algorithms on directed and weighted graphs with overlapping communities. *Phys. Rev. E* **80**, 016118 (2009).

[4] Rand, W. M. Objective criteria for the evaluation of clustering methods. *J Am Stat Assoc* **66**, 846–850 (1971).

[5] Decelle, A., Krzakala, F., Moore, C. & Zdeborová, L. Asymptotic analysis of the stochastic block model for modular networks and its algorithmic applications. *Phys. Rev. E* **84**, 066106 (2011).

[6] Massoulié, L. Community detection thresholds and the weak Ramanujan property. In *Proceedings of the Forty-Sixth Annual ACM Symposium on Theory of Computing*, STOC '14, 694–703 (Association for Computing Machinery, New York, NY, USA, 2014).

[7] Chung, F. R. K. *Spectral Graph Theory*, vol. 92 (American Mathematical Soc., 1997).

[8] Girvan, M. & Newman, M. E. J. Community structure in social and biological networks. *Proc. Natl. Acad. Sci. U.S.A.* **99**, 7821–7826 (2002).

[9] Blondel, V. D., Guillaume, J.-L., Lambiotte, R. & Lefebvre, E. Fast unfolding of communities in large networks. *J. Stat. Mech. Theory Exp.* **2008**, P10008 (2008).

[10] Ni, C.-C., Lin, Y.-Y., Luo, F. & Gao, J. Community detection on networks with Ricci flow. *Sci. Rep.* **9**, 9984 (2019).

[11] Delvenne, J.-C., Yaliraki, S. N. & Barahona, M. Stability of graph communities across time scales. *Proc. Natl. Acad. Sci. U.S.A.* **107**, 12755–12760 (2010).

[12] Tremblay, N. & Borgnat, P. Graph wavelets for multiscale community mining. *IEEE Trans. Signal Process.* **62**, 5227–5239 (2014).

[13] Cormen, T. H., Leiserson, C. E., Rivest, R. L. & Stein, C. *Introduction to Algorithms, Third Edition* (The MIT Press, 2009), 3rd edn.

[14] Al-Mohy, A. H. & Higham, N. J. A new scaling and squaring algorithm for the matrix exponential. *SIAM J. Matrix Anal. Appl.* **31**, 970–989 (2010).

[15] Lee, Y. T. & Sidford, A. Path finding methods for linear programming: Solving linear programs in Õ(vrank) iterations and faster algorithms for maximum flow. In *2014 IEEE 55th Annual Symposium on Foundations of Computer Science*, 424–433 (2014).

[16] Cuturi, M. Sinkhorn distances: Lightspeed computation of optimal transport. In Burges, C. J. C., Bottou, L., Welling, M., Ghahramani, Z. & Weinberger, K. Q. (eds.) *Adv. Neural. Inf.. Process. Syst. 26*, 2292–2300 (Curran Associates, Inc., 2013).

[17] Flamary, R. & Courty, N. Pot python optimal transport library (2017). URL https://pythonot.github.io/.

[18] Zhang, P. & Moore, C. Scalable detection of statistically significant communities and hierarchies, using message passing for modularity. *Proc. Natl. Acad. Sci. U.S.A.* **111**, 18144–18149 (2014).

[19] Rosvall, M. & Bergstrom, C. T. Maps of random walks on complex networks reveal community structure. *Proc. Natl. Acad. Sci. U.S.A.* **105**, 1118–1123 (2008).

[20] Reichardt, J. & Bornholdt, S. Statistical mechanics of community detection. *Phys. Rev. E* **74**, 016110 (2006).

[21] Enright, A. J., Van Dongen, S. & Ouzounis, C. A. An efficient algorithm for large-scale detection of protein families. *Nucleic Acids Res* **30**, 1575–1584 (2002).

[22] Baumes, J., Goldberg, M. & Magdon-Ismail, M. Efficient identification of overlapping communities. In *International Conference on Intelligence and Security Informatics*, 27–36 (Springer, 2005).

[23] Raghavan, U. N., Albert, R. & Kumara, S. Near linear time algorithm to detect community structures in large-scale networks. *Phys. Rev. E* **76**, 036106 (2007).

[24] Pons, P. & Latapy, M. Computing communities in large networks using random walks. In *J. Graph Algorithms Appl.* (Citeseer, 2006).

[25] Li, P.-Z., Huang, L., Wang, C.-D. & Lai, J.-H. Edmot: An edge enhancement approach for motif-aware community detection. In *Proceedings of the 25th ACM SIGKDD International Conference on Knowledge Discovery & Data Mining*, 479–487 (2019).

[26] Wang, X. *et al.* Community preserving network embedding. In *Proceedings of the Thirty-First AAAI Conference on Artificial Intelligence*, AAAI'17, 203–209 (AAAI Press, 2017).

[27] Clauset, A., Newman, M. E. & Moore, C. Finding community structure in very large networks. *Phys. Rev. E* **70**, 066111 (2004).
